# Consumer behavior and awareness regarding products for food hypersensitivity: trends, motivations, and educational gaps

**DOI:** 10.3389/fnut.2025.1576403

**Published:** 2025-05-08

**Authors:** Agata Kiciak, Aleksandra Kołodziejczyk, Natalia Kuczka, Paulina Łokaj, Wiktoria Staśkiewicz-Bartecka, Agnieszka Białek-Dratwa, Agnieszka Bielaszka, Daria Dobkowska-Szefer, Oskar Kowalski, Marek Kardas

**Affiliations:** ^1^Department of Food Technology and Quality Assessment, Department of Dietetics, Faculty of Public Health in Bytom, Medical University of Silesia in Katowice, Bytom, Poland; ^2^Department Human Nutrition, Department of Dietetics, Faculty of Public Health in Bytom, Medical University of Silesia in Katowice, Bytom, Poland

**Keywords:** food allergies, food intolerances, food hypersensitivity, lactose free products, gluten free products

## Abstract

**Background/Objectives:**

Food hypersensitivity, including allergies and intolerances, represents a significant and growing health challenge. To address this issue effectively, it is essential to implement tailored dietary interventions that can effectively reduce symptoms and improve patients' quality of life. The study aimed to analyze the consumption of specialist products intended for people with food hypersensitivities and to assess knowledge of food allergies and food intolerances.

**Methods:**

The study was conducted from January to May 2021 using an original survey questionnaire that was made available to respondents in electronic form. The study involved 191 people, including 132 women and 59 men.

**Results:**

The analysis showed that the knowledge of respondents about the labeling of products dedicated to people with food allergies and intolerances was generally satisfactory, but significant gaps were found in the knowledge of less common allergens, such as histamine or egg white. In addition, the consumption of products dedicated to food allergies and intolerances was comparable in the groups of diagnosed and healthy people.

**Conclusions:**

Although the general knowledge of consumers about product labels was high, there are significant gaps in knowledge of more specific allergens. Moreover, the results suggest that products dedicated to people with food allergies and intolerances are consumed by both healthy and diagnosed people, which indicates the need for further education in this area.

## 1 Introduction

Food hypersensitivity, which encompasses a broad spectrum of bodily reactions to substances present in food that do not elicit undesirable symptoms in healthy individuals, is becoming an increasing challenge in the realm of public health (1–4). Its incidence is on the rise, driven by various environmental factors and lifestyle changes (1, 2, 5, 6). Food hypersensitivities primarily include food allergies and intolerances, often referred to as undesirable non-toxic reactions, which distinguishes them from responses caused by consuming toxic substances. Despite their non-toxic nature, they can cause a range of troublesome clinical symptoms and significantly affect patients' quality of life (6).

The main method for managing the health of individuals with food hypersensitivity is an elimination diet, which requires excluding products that trigger clinical reactions (7). Effective dietary treatment hinges on correctly identifying the allergen or intolerant substance, thereby reducing symptoms and improving patients' quality of life (8). In response to the growing demand for safe food tailored to the needs of this group of consumers, the functional food market is expanding rapidly, offering a wide range of products free from common allergens such as gluten and lactose (9–11). In addition to their nutritional value, these products aim to mitigate ailments caused by food hypersensitivity, thus offering benefits that go beyond standard nutritional functions (6, 11).

A significant increase in interest is particularly evident in gluten-free and lactose-free products, which cater to individuals with food intolerances, allowing them to follow an elimination diet without sacrificing dietary diversity (10–12). The well-developed gluten-free product market includes both ready-made items and semi-finished products that enable the preparation of varied meals. These products may be entirely gluten-free or contain trace amounts of gluten, consistent with current regulatory requirements (13). Similarly, the range of “lactose-free” products is expanding rapidly, produced through various technological processes—such as enzymatic, membrane, or chromatographic methods—to remove or significantly reduce lactose content (14, 15). Despite the wide availability of gluten-free and lactose-free products, the market for foods dedicated to other allergies remains limited due to the diversity of allergens and the complex mechanisms underlying allergic reactions. In these cases, individually tailored elimination diets are recommended, making it challenging to create standardized products for a broader consumer base (16).

The aim of this study was to analyze the consumption of specialized products intended for individuals with food hypersensitivities. An additional objective was to determine the extent to which consumers' choices regarding these products are driven by genuine health needs—stemming from the necessity to alleviate symptoms of food allergies and intolerances—vs. prevailing dietary trends, such as the growing popularity of gluten-free and lactose-free foods. Beyond identifying consumers' motivations, the study also sought to assess the factors influencing their dietary choices, including the level of knowledge about products designed for people with food hypersensitivities, and to understand the role of health education in shaping these choices.

## 2 Materials and methods

### 2.1 Project study

The research was carried out between January and May 2021 using an original survey questionnaire administered through the CAWI (Computer-Assisted Web Interview) method. This approach, widely recognized in behavioral research, was selected for its efficiency, extensive reach, and ability to ensure respondent anonymity. Data collection was conducted via the Google Forms platform, chosen for its ease of use, accessibility, and automated data aggregation features, facilitating streamlined response analysis. Participants received a QR code after their medical appointment, which directed them to the survey.

The study protocol (PCN/0022/KB/299/19/20, date of approval: 29 January 2020) was reviewed by the Bioethics Committee of the Silesian Medical University in Katowice and was approved. The Declaration of Helsinki of the World Medical Association guided the conduct of this study. Each person participating in the study gave informed consent to participate in the study and was informed about the anonymity of the results.

### 2.2 Participants

The study involved 191 participants, including 132 women (69.1%) and 59 men (30.9%). All participants were patients of a gastroenterology clinic located in the city of Katowice, Poland. This setting was selected to ensure access to individuals potentially experiencing dietary challenges and hypersensitivity-related issues, allowing for a focused and relevant analysis of the target population.

The inclusion criteria for participation were as follows: (1) completion of at least 18 years of age, (2) registered patient status at the gastroenterology clinic, (3) attendance at a medical appointment during the study period, and (4) providing informed consent to participate in the research.

Participation in the study was voluntary, with anonymity guaranteed to respondents. All respondents provided informed consent for their data to be used solely for scientific purposes, ensuring ethical compliance and encouraging honest and accurate responses.

### 2.3 Research tools

The questionnaire consisted of 25 questions divided into two key sections. The first section focused on sociodemographic information, collecting data on respondents' age, gender, education level, body weight, and height. The second section was dedicated to examining issues related to food allergies, intolerances, and the respondents' experiences with products designed for individuals with these conditions. To provide comprehensive insights, the questionnaire included a combination of open-ended questions, allowing for detailed written responses, and closed-ended questions, offering single or multiple-choice options for structured data collection. The questions in this section were carefully selected and validated by a team of experts in the fields of nutrition, public health, and behavioral research to ensure their relevance and accuracy.

Before the main study commenced, a pilot study was conducted to assess the clarity, structure, and overall effectiveness of the questionnaire. This phase involved 20 participants (12 women and eight men), who provided feedback on the survey's design and comprehensibility. Based on the suggestions and observations gathered during the pilot study, adjustments were made to improve the clarity and usability of the questionnaire. After these refinements, the finalized version was deployed electronically for the main study, ensuring a high level of consistency and ease of participation.

The nutritional status of the respondents was assessed based on the body mass index (BMI), calculated in accordance with the recommendations of the World Health Organization (WHO). BMI was calculated using the formula (17):


(1) $$\begin{array}{l} BMI=\frac{body\;weight\;\left[kg\right]}{{\left(height[m]\right)}^{2}} \end{array}$$

Following the assumptions of the WHO, the following ranges of BMI values were adopted (17):

- <18.5—underweight
- 18.5–24.9—normal,
- 25.0–29.9—overweight,
- 30.0–34.9—obesity class I,
- 35.0–39.9—obesity class II,
- >40.0—obesity class III.

Participation in the study was voluntary, and respondents were informed about the anonymity of the study and the use of its results only for scientific purposes.

### 2.4 Statistical analysis

Statistical analyses were performed using Statistica v.13.3 (Stat Soft Poland) and the R package v. 4.0.0 (2020) under the GNU GPL (The R Foundation for Statistical Computing).

To present quantitative data, mean values and standard deviations (X±S) were calculated; for qualitative data, percentage notation was used. Associations between nominal variables were examined using Fisher's exact test and the Chi-square (χ^2^) test. The analysis also included cross-tabulations to examine relationships between variables such as education level and knowledge of food labels, as well as between dietary symptoms and specific food categories.

The criterion of statistical significance was *p* < 0.05.

## 3 Results

The study involved 191 participants, of whom 132 were women (69.1%) and 59 were men (30.9%). The largest age group was made up of respondents aged 19–29, comprising 63.9% of the study sample. Based on the calculated BMI of each respondent, participants were classified into appropriate ranges according to the criteria of the World Health Organization (WHO). The results showed that 63.9% of respondents were within the range of body weight considered normal, 20.4% had a BMI value indicating overweight, while the remaining people were obese or underweight. In terms of education level, the largest group were participants with tertiary education (51.8%), followed by people with secondary education (23%), vocational education (20.9%) and primary education (4.2%). The above results are presented in Table 1.

**Table 1:** Sociodemographic characteristics of respondents (*N* = 191).

**Characteristics of the respondents**	**Category**	***N* (%)**
Gender	Women	132 (69.1)
	Men	59 (30.9)
Age [years]	≤18	8 (4.2)
	19–29	122 (63.9)
	30–39	44 (23)
	40–50	7 (3.7)
	≥50	10 (5.2)
BMI [kg/m^2^]	Underweight	10 (5.2)
	Normal weight	122 (63.9)
	Overweight	39 (20.4)
	Obesity class I	18 (9.4)
	Obesity class III	2 (1.1)
Education	Primary	8 (4.2)
	Vocational	40 (21)
	Secondary	44 (23)
	Tertiary	99 (51.8)

In the study group, 68.1% of respondents declared no chronic diseases, while among people with chronic diseases thyroid disorders (17.8%) and insulin resistance (10.5%) dominated. From the analysis of the responses of all respondents, it should be noted that 54.7% of respondents did not follow any diet, while in the group of people with chronic diseases 44.1% declared following a diet, and in the group of healthy people 37.9%. Statistical analysis showed that the occurrence of chronic diseases had no significant effect on the use of a diet (*p* = 0.957). In relation to digestive complaints, 37.2% of participants did not report any symptoms after consuming food products, while among the remaining most frequently reported symptoms were bloating (43.5%), abdominal pain (38.7%) and diarrhea (36.1%). In response to the question about products causing food ailments, most people indicated milk and dairy products (49.2%), as well as vegetables (11.5%) and fruit (8.9%).%). In relation to the occurrence of food allergies and intolerances, 21.5% of respondents declared a diagnosed food allergy, 22.5% food intolerance, while 56% of the respondents did not report any of these ailments. Among people with food allergies or intolerances, 67.9% (*N* = 57) used an elimination diet, 23.8% (*N* = 20) a rotation diet, and 7 people did not use any diet. However, none of the participants used desensitization. People with diagnosed food intolerance significantly more often (*p* = 0.0004) consumed products dedicated to people with gluten intolerance compared to people in whom food intolerance was not diagnosed. In the group of people with diagnosed intolerance, 48.8% consumed these products several times a week, while in the group without diagnosis, this percentage was 38.5%. The consumption of products dedicated to lactose intolerance did not differ significantly between people with diagnosed food intolerance and those without a confirmed diagnosis (*p* = 0.285). In the case of food allergy, statistical analysis showed a significant difference in the frequency of consumption of products dedicated to lactose intolerance, where people with diagnosed food allergy consumed them more often than people without a diagnosis of allergy (*p* = 0.003). It should be emphasized, however, that despite significant differences, the largest percentage of respondents indicated that they did not consume products dedicated to lactose intolerance. Comparative analysis of the consumption of products dedicated to gluten intolerance did not show significant differences in the frequency of consumption of the selected product category between people with diagnosed and undiagnosed food allergy. The results are presented in Table 2.

**Table 2:** Frequency of consumption of products dedicated to selected intolerances, including the diagnosis of food allergy and intolerance.

**Consumption of dedicated products in case of intolerance**	**Group of subjects**		**Frequency of consumption of the selected product category (%)**						***p*-value**
			**Several times a day**	**Once a day**	**Several times a week**	**Once a week**	**Several times a month**	**Never**	
Gluten	Food intolerance	Undiagnosed (*N* = 148)	1.4	6.8	38.5	5.4	23.6	24.3	0.0004^*^
		Diagnosed (*N* = 43)	23.3	0	48.8	0	9.3	18.6	
	Food allergy	Undiagnosed (*N* = 150)	6.7	6.7	36	5.3	21.3	24	0.646
		Diagnosed (*N* = 41)	4.9	0	58.5	0	17.07	19.5	
Lactose	Food intolerance	Undiagnosed (*N* = 148)	9.5	15.5	25.7	2	17.6	29.1	0.285
		Diagnosed (*N* = 43)	18.6	0	18.6	0	11.6	51.2	
	Food allergy	Undiagnosed (*N* = 150)	9.3	8	30.7	0.7	16	35.3	0.003^*^
		Diagnosed (*N* = 41)	19.5	26.8	0	4.9	17.1	29.3	

* p < 0.05.

Analysis of the frequency of consumption of selected food products in the study group showed a varied trend in the consumption of individual food groups. Milk and dairy products were consumed several times a week by 35.6% of respondents, while 17.8% of respondents completely excluded them from their diet. Chicken eggs were consumed by 55.49% of the study participants 2–3 times a week, and 12% of respondents declared their complete elimination. Nuts were consumed by 51.3% of people several times a month or week, while 17.8% of respondents completely excluded them from their diet. In relation to pickled products, 53.9% of respondents declared their consumption several times a month, and 2.1% completely excluded them from their diet. Blue cheese and long-ripened cheese were eliminated from the diet by 52.4% of respondents, while 36.6% consumed them several times a month. Fish and seafood were consumed several times a month by 60.7% of the study participants, and 8.4% eliminated them completely. Detailed results are presented in Table 3.

**Table 3:** Frequency of consumption of selected food products in the study group (*N* = 191).

**Product group**	**Consumption frequency N (%)**					
	**Several times a day**	**Once a day**	**Several times a week**	**Once a week**	**Several times a month**	**Never**
Milk and dairy products	28 (14.7)	38 (19.9)	68 (35.6)	14 (7.3)	9 (4.7)	34 (17.8)
Nuts	2 (1)	8 (4.2)	38 (19.9)	11 (5.8)	98 (51.3)	34 (17.8)
Pickled products	0 (0)	3 (1.6)	57 (29.8)	24 (12.6)	103 (53.9)	4 (2.1)
Blue and long-ripened cheeses	0 (0)	5 (2.6)	12 (6.3)	4 (2.1)	70 (36.6)	100 (52.4)
Fish and seafood	0 (0)	2 (1.1)	10 (5.2)	57 (29.8)	116 (60.7)	6 (8.4)

**Figure 1:**
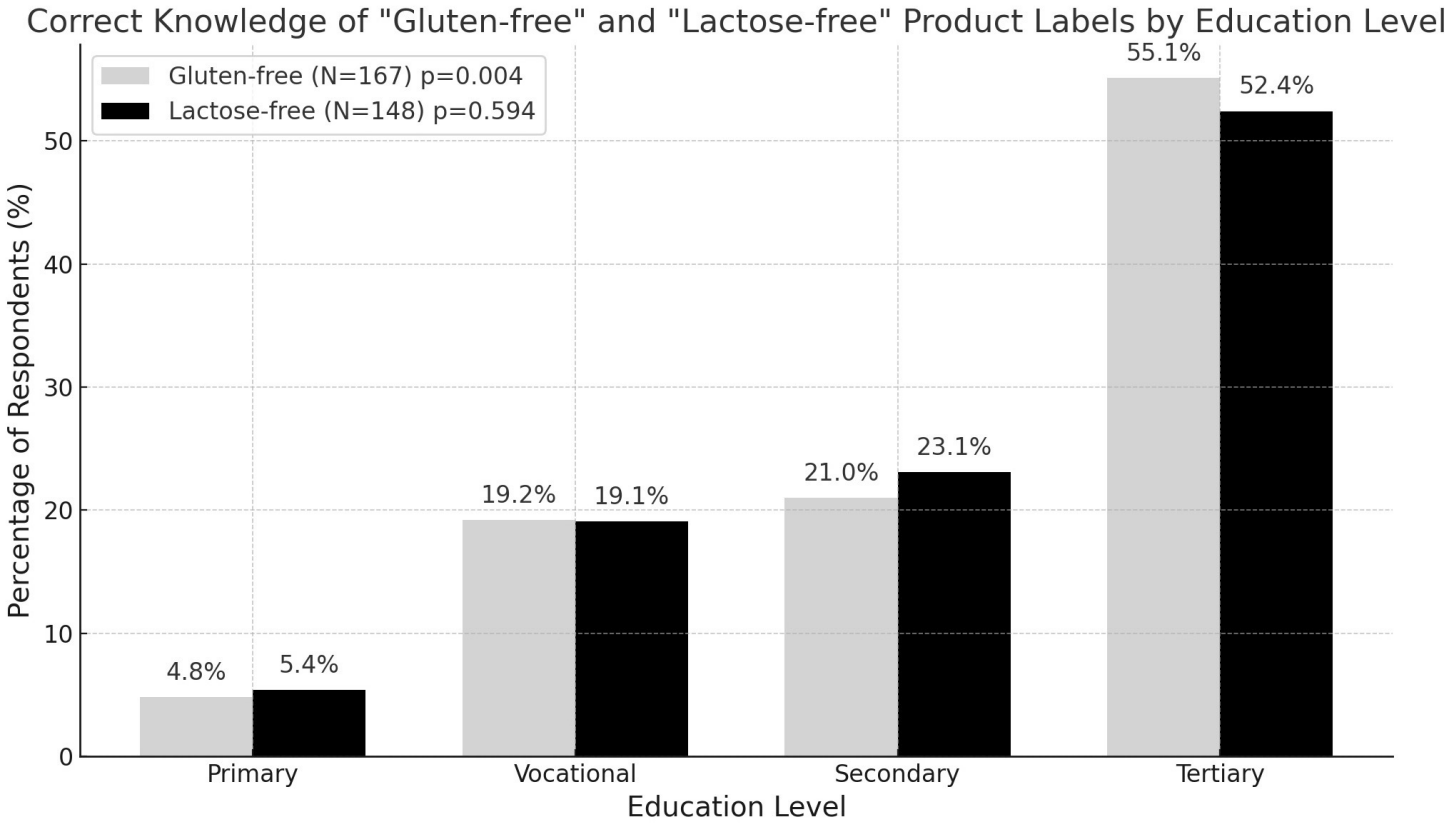
Correct knowledge of selected product labels, taking into account education.

The analysis of knowledge of product labels dedicated to selected food intolerances showed that people with tertiary education were significantly more likely (*p* = 0.004) to correctly identify the labels of “gluten-free” products than those with a lower level of education. In the group of respondents who correctly recognized the “gluten-free” labels, 55.1% were people with tertiary education. In the group of respondents with tertiary education, 92.9% (*N* = 92) gave the correct answer. In the groups of people with secondary, vocational and primary education, the percentage of correct answers was 79.6% (*N* = 35), 80% (*N* = 32) and 100% (*N* = 8), respectively. In relation to the “lactose-free” labels, the level of education did not show a significant effect on the correctness of the answer—people with different levels of education identified the correct labels to a similar extent. The above results are presented in Figure 1.

In relation to knowledge of products dedicated to food allergies and intolerances, respondents provided answers indicating preferences regarding product groups that should be included in the diet of people with specific hypersensitivities. The largest number of respondents indicated milk and dairy products (75.4%) and cereal products (55%) as products dedicated to food allergies and intolerances. A smaller percentage of respondents considered vegetables (14.7%) and fruits (15.2%) as products requiring special consideration, and the fewest people (10.5%) indicated sweets and snacks.

In the context of eliminating products in the case of food intolerances, respondents most often indicated wheat products (59.7%) and bread (23%) in the case of gluten intolerance. For lactose intolerance, milk (43.5%) and yogurts (17.3%) were most often mentioned, and some respondents referred to them generally as “dairy products” (29.8%). In the case of cow's milk protein allergy, most respondents (58.1%) indicated milk, and 27.8% indicated dairy products. In the case of egg protein allergy, 42.4% indicated chicken eggs, and 22% believed that egg-based products should be eliminated. In the case of histamine allergy, the vast majority (72.3%) of respondents did not indicate specific products to eliminate, and among those who provided an answer, the most frequently mentioned were fish and seafood (18.3%) and canned food (13.6%) (Table 4).

**Table 4:** Foods to be avoided in different types of food hypersensitivity (*N* = 191).

**Type of hypersensitivity**	**Product/product group**	**Number of indications N (%)**
Gluten intolerance	Wheat products	114 (59.69)
	Bread	44 (23.04)
	Pasta	25 (13.09)
	I don't know	65 (34.03)
Lactose intolerance	Milk	83 (43.46)
	Dairy	57 (29.84)
	Yogurts	33 (17.28)
	I don't know	23 (12.04)
Cow's milk protein allergy	Milk	111 (58.12)
	Dairy products	53 (27.75)
	I don't know	58 (30.37)
Hen's egg protein allergy	Hen's eggs	81 (43.41)
	Egg-based products	42 (21.99)
	I don't know	73 (38.22)
Histamine allergy	Fish and seafood	35 (18.32)
	Canned foods	26 (13.61)
	Nuts	23 (12.04)
	Mold-ripened cheeses	20 (10.47)
	I don't know	138 (72.25)

## 4 Discussion

The results of the analysis of digestive complaints in the study group show that the vast majority of respondents (68.2%) experience various stomach symptoms, including bloating (43.5%), abdominal pain (38.7%) and diarrhea (36.1%). These data are consistent with the results of studies conducted in America, where 61% of respondents reported the occurrence of stomach symptoms in the past week (18). Among the reported symptoms in the American study, the most frequently indicated were heartburn/reflux (30.9%), abdominal pain (24.8%), bloating (20.6%), diarrhea (20.2%) and constipation (19.7%) (18). The similarities in the results suggest that the discussed complaints are common, regardless of the region. It is worth noting that in the study group, respondents most often indicated milk and dairy products (49.2%) as products causing digestive symptoms, which may suggest that frequent stomach complaints are related to lactose intolerance. Lactose intolerance is a common health problem worldwide, affecting 57% to 70% of the population, and in Europe it occurs in about 28% of people (19–21). The high percentage of respondents indicating milk and dairy products as the cause of stomach problems confirms the importance of dairy products as potential sources of food symptoms and indicates the need for extensive education in the field of recognizing lactose intolerance and the need to eliminate or limit milk and dairy products in the diet of people showing symptoms of food hypersensitivity.

In the context of food allergies and intolerances, 21.5% of respondents declared the occurrence of food allergy, and 22.5% food intolerance, which is similar to the results of European studies, which estimated that about 19.9% of the population struggles with various food allergies (22). In response to these conditions, the elimination diet turned out to be the most commonly used treatment method, used by 67.86% of people suffering from food allergies or intolerances in the study group. Elimination diet is the basis of treatment in food hypersensitivity, which was also confirmed by other authors (4, 7, 8, 23). The most frequently eliminated products from the diet of respondents were long-ripened cheeses (52.4%), milk and dairy products (17.8%), nuts (17.8%), chicken eggs (12%) and fish and seafood (3.1%). These results partially coincide with the research of Lim et al., who showed that the most frequently restricted food among the examined people with inflammatory bowel disease was milk and dairy products (32.7%) and raw fish (24.5%) (24). The use of an elimination diet is associated with the risk of nutrient deficiencies, especially if the eliminated products are not properly replaced with their nutritional equivalents, which may lead to problems with ensuring an adequate level of protein and other macronutrients (25). As studies emphasize, an extended elimination diet requires strict control to prevent impaired nutrition and related health complications (25). Therefore, it is necessary to ensure proper monitoring of the nutritional status of people using elimination diets, which is crucial for minimizing the risk of nutritional deficiencies and improving the overall health and quality of life of these patients.

The conducted studies showed significant differences in the frequency of consumption of products dedicated to gluten intolerance between the groups of respondents with diagnosed and undiagnosed food intolerance. The consumption of gluten-free products was declared by 81.4% of people with diagnosed food intolerance and 75.68% of people without a diagnosis, which indicates the common use of these products regardless of health condition. These results are consistent with the observations of Alencar et al., who indicated that as many as 93% of people with diagnosed gluten intolerance follow a strict gluten-free diet, which is associated with the regular use of specialist products (26). At the same time, the high percent-age of healthy people using gluten-free products may be the result of the growing popularity of the gluten-free diet as a supposedly healthier alternative, which is also emphasized by Niland et al., who point to the dynamic growth in sales of this type of products (9). Nevertheless, the authors emphasize that eliminating gluten without medical indications may be associated with adverse health effects, such as nutrient deficiencies (9). Similar results were observed for lactose-free products, which were consumed by 70.73% of people with diagnosed food allergies and 64.67% of people without a diagnosis. These results are consistent with the data of Szabó et al. (27), where 66.7% of people sensitive to lactose regularly used this type of products, which confirms their important role in the daily diet of people with food hypersensitivity. The obtained results indicate the importance of products dedicated to elimination diets, both in the health context and as an element of consumer preferences among a wide group of recipients.

The results of the conducted studies showed significant differences in the ability to recognize the labels of food products intended for people with selected food intolerances, taking into account the level of education of the respondents. According to the analysis conducted by Mikołajczak, most of the labels of gluten-free products met the applicable legal requirements, which contributes to their correct reception by consumers (28). Similar conclusions were drawn by Krasnowska and Salejda (29), who noted that the way of presenting information on the packaging is understandable for Polish consumers. The results of our own study showed that most respondents correctly identified the labels “gluten-free” and “lactose-free,” with people with tertiary education significantly more often (p=0.004) recognizing the “gluten-free” labels than people with a lower level of education. In the case of “lactose-free” labels, no effect of the level of education was observed on the correctness of the answer, which suggests that this type of information is more common and recognizable regardless of education. The high correctness of the answer (100%) in the group of people with primary education may be related to the small size of this group, which limits the possibility of generalizing the results. Current labeling standards seem to be effective in ensuring a high level of recognition of products intended for people with food intolerances. Further educational activities may, however, contribute to increasing consumer awareness and their confidence in correctly identifying dedicated products.

As consumers' nutritional awareness increases, there is a growing need to deepen their knowledge of food allergies and intolerances (30). The analysis revealed significant gaps in this area, as a significant proportion of respondents did not have sufficient knowledge of allergens contained in food. In the case of hypersensitivity to histamine, egg white and cow's milk protein, 72.3%, 38.2% and 30.4% of respondents, respectively, did not know which products should be eliminated from the diet. Furthermore, respondents were often able to indicate only a few products that should be eliminated from the diet, which suggests insufficient knowledge of the full range of allergens. Although the percentage of correct answers was higher for gluten and lactose intolerance, there were still errors and imprecisions, which indicates limited consumer awareness of available products for people with food allergies. Similar limitations in knowledge about food allergies were observed in the studies by Wieser et al. (31) and Shafie and Azman (32), who noted that a large proportion of people working with food have only a moderate level of knowledge about allergens. On the other hand, the studies by Lee and Sozen (33) indicate that employees of the catering industry in the United States are characterized by a good level of knowledge about food allergies, which may result from their professional contact with food. These differences may result from different groups of respondents, in which people professionally associated with the food sector should have a higher awareness of allergens compared to people not associated with this industry. In light of these results, an important step in improving the state of nutritional awareness of the society is to implement educational activities that will enable consumers to make informed food choices and effectively avoid ingredients that may cause allergic reactions.

In recent investigations, consumer understanding of food allergen labeling and the effectiveness of dietary interventions have been rigorously examined. For instance, Gupta et al. found that clear labeling significantly improves consumer comprehension and facilitates safer dietary practices, although gaps remain regarding less common allergens (34). Similarly, Brown and Ping reported that stringent labeling standards combined with targeted consumer education enhance the correct identification of food allergens, thereby contributing to better dietary management (35). Additionally, Simons et al. demonstrated that integrated public health campaigns, alongside advances in food labeling technology, can further improve consumer awareness and mitigate adverse outcomes related to food hypersensitivity (36). These findings underscore the importance of comprehensive strategies that combine improved labeling practices with robust educational initiatives, reinforcing the implications of our own study in this evolving field.

Based on the results of our study, as well as comparison with international literature, it can be concluded that although awareness of food allergies and intolerances is increasing, there are still many areas in which further public education is required, especially in the context of the use of appropriate elimination diets.

### 4.1 Strengths and limitations

The study has several notable strengths that contribute to the robustness of its findings. Targeting patients from a gastroenterology clinic ensured a highly relevant sample group, as these individuals are more likely to experience food hypersensitivity issues. The use of a comprehensive questionnaire, combining open-ended and closed-ended questions, allowed for the collection of detailed and nuanced data on consumer behaviors and motivations. Conducting a pilot study prior to the main research enhanced the reliability and clarity of the questionnaire, ensuring the collection of high-quality data. Additionally, the identification of significant knowledge gaps among respondents provides valuable insights for designing targeted educational programs aimed at improving awareness of food hypersensitivity and elimination diets.

However, the study also has limitations that should be acknowledged. The sample drawn from a single gastroenterology clinic in Katowice, limits the generalizability of the findings to broader populations or other regions. The reliance on self-reported data introduces the potential for recall or reporting bias, which could affect the accuracy of the results. Furthermore, the study focused primarily on common intolerances such as gluten and lactose, offering less insight into rarer hypersensitivities like histamine intolerance. The cross-sectional design precludes the ability to observe changes over time or establish causal relationships. These limitations underscore the need for further research to validate and expand on these findings across larger and more diverse populations.

## 5 Conclusions

The results of the conducted studies indicate a satisfactory level of knowledge of the respondents regarding the labeling of selected food products dedicated to people with food allergies and intolerances, which indicates high consumer awareness in the area of identifying “gluten-free” and “lactose-free” products. However, the analysis revealed significant gaps in consumer knowledge regarding food allergies and intolerances, especially in relation to less common allergens, such as histamine or egg white. Importantly, products dedicated to food allergies and intolerances are consumed comparably often by people with diagnosed food hypersensitivity, as well as by healthy people, which may indicate a strong influence of current dietary trends, such as the fashion for a gluten-free or lactose-free diet. Therefore, although the general knowledge of product labeling is at a good level, there is a need to intensify educational activities in the area of allergens and their impact on health, which will allow for making more informed dietary decisions, especially among undiagnosed people.

## Data Availability

The original contributions presented in the study are included in the article/Supplementary material, further inquiries can be directed to the corresponding authors.
