# Supplementary material for: Consumer behavior and awareness regarding products for food hypersensitivity: trends, motivations, and educational gaps
Source: Front Nutr. 2025 May 8;12:1576403. doi: 10.3389/fnut.2025.1576403 (PMC12094956; doi:10.3389/fnut.2025.1576403)
Supplement: Supplementary file 1 [file Data_Sheet_1.pdf]

Dear Sir or Madam,

I kindly invite you to participate in a scientific study concerning the "Assessment of consumption and assortment of products intended for people with allergies and food intolerances." The aim of this study is to analyze dietary habits and the availability of food products dedicated to individuals with food sensitivities, contributing to enhancing knowledge in the field of nutrition and public health.

The survey is anonymous, and the obtained data will be used solely for scientific purposes, in accordance with applicable ethical standards and data protection principles.

The questionnaire consists of 25 questions, including both closed-ended (single and multiple choice) and open-ended questions. To ensure the reliability of the results, please carefully read the questions and provide answers that reflect your actual situation.

Your participation in this study represents a valuable contribution to the development of scientific knowledge and may help improve the market offer to meet the needs of people with allergies and food intolerances.

Sincerely,

Part I

1. Gender

- Female
- Male

2. Age (in years)..............................
3. Weight (kg)..............................
4. Height (cm)..............................
5. Education Level

- Primary
- Vocational
- Secondary
- Tertiary

Part II

1. Do you have any chronic diseases or other health issues? (You can select multiple answers)

- No, I am healthy
- Diabetes
- Insulin resistance
- Hypertension
- Hypercholesterolemia
- Thyroid diseases
- Celiac disease

- Other (please specify): ....

2. After consuming certain food products, have you noticed the occurrence of symptoms such as: (You can select multiple answers)

- Abdominal bloating
- Stomach pain
- Diarrhea
- Nausea
- Vomiting
- Itching
- Sneezing
- Dry cough
- I have not noticed any of the above symptoms after consuming food products (proceed to question 4)

3. What food products caused the symptoms mentioned above? ....
4. Have you been diagnosed with a food allergy?

- Yes
- No (proceed to question 6)

5. How was the food allergy diagnosed? (You can select multiple answers)

- By a doctor based on medical history
- By a doctor based on physical examination
- By a doctor based on a provocation test
- By a doctor based on skin test results
- The food allergy was not diagnosed by a doctor, only based on personal observation of the body's reactions
- Other (please specify): ....

6. Have you been diagnosed with food intolerance?

- Yes
- No (proceed to question 9)

7. How was the food intolerance diagnosed? (You can select multiple answers)

- By a doctor based on medical history
- By a doctor based on IgG allergen panel results
- Food intolerance was not diagnosed by a doctor, only based on personal observation of the body's reactions
- Other (please specify): ....

8. As part of the treatment of food allergies/intolerances, do you follow:

- An elimination diet that completely excludes the allergen from the diet
- A rotation diet based on alternating consumption of tolerated and harmful foods
- Desensitization

- Other (please specify): ....

9. Do you follow any particular diet? (You can select multiple answers)

- Vegetarian
- Vegan
- Low-calorie
- Reduced carbohydrate intake
- Easily digestible
- High-fiber
- High-protein
- Low-protein
- Reduced fat intake
- I do not follow any diet
- Other (please specify): ....

10. List the products that should be eliminated from the diet of a person with gluten intolerance: ....
11. List the products that should be eliminated from the diet of a person with lactose intolerance: ....
12. List the products that should be eliminated from the diet of a person allergic to cow's milk protein: ....
13. List the products that should be eliminated from the diet of a person allergic to egg protein: ....
14. List the products that should be eliminated from the diet of a person allergic to histamine: ....
15. In your opinion, are products dedicated to allergies and food intolerances part of the group of: (You can select multiple answers)

- Dairy products
- Meat and fish products
- Cereal products
- Fats
- Sweets, desserts, salty snacks
- Vegetables
- Fruits
- Nuts and seeds

16. How often do you consume products intended for gluten intolerance (labeled as 'gluten-free')?

- Several times a day
- Once a day
- Several times a week
- Once a week
- Several times a month
- Never

17. How often do you consume products intended for lactose intolerance (labeled as 'lactose-free')?

- Several times a day
- Once a day
- Several times a week
- Once a week
- Several times a month
- Never

18. How often do you consume milk and dairy products?

- Several times a day
- Once a day
- Several times a week
- Once a week
- Several times a month
- I have completely eliminated dairy products from my diet

19. How often do you consume chicken eggs?

- Every day
- 2–3 times a week
- Once a week
- Several times a month
- I have completely eliminated chicken eggs from my diet

20. How often do you consume nuts?

- Several times a day
- Once a day
- Several times a week
- Once a week
- Several times a month
- I have completely eliminated nuts from my diet

21. How often do you consume fermented products?

- Several times a day
- Once a day
- Several times a week
- Once a week
- Several times a month
- I have completely eliminated fermented products from my diet

22. How often do you consume mold-ripened and aged cheeses?

- Several times a day
- Once a day
- Several times a week
- Once a week
- Several times a month
- I have completely eliminated mold-ripened and aged cheeses from my diet

23. How often do you consume fish and seafood?

- Every day
- 2–3 times a week
- Once a week
- Several times a month
- I have completely eliminated fish and seafood from my diet

24. Do you know how gluten-free products should be labeled?

- The label should include the description "gluten-free product," and the manufacturer may also mark it with the "crossed-out ear" symbol licensed by AOECS.
- The manufacturer should use any symbol resembling the one licensed by AOECS.
- No manufacturer has the right to label products as gluten-free; the absence of gluten should be evident from the product composition.

25. Do you know how lactose-free products should be labeled?

- There is no requirement to label lactose-free products.
- The label should include a "crossed-out cow" symbol in a visible place.
- There is no specific symbol for labeling lactose-free products; such information can be provided by the manufacturer following general food labeling laws.
